# Machine Learning-Based Estimation of Knee Joint Mechanics from Kinematic and Neuromuscular Inputs: A Proof-of-Concept Using the CAMS-Knee Datasets

**DOI:** 10.3390/bioengineering13020173

**Published:** 2026-01-31

**Authors:** Yara N. Derungs, Martin Bertsch, Kushal Malla, Allan Maas, Thomas M. Grupp, Adam Trepczynski, Philipp Damm, Seyyed Hamed Hosseini Nasab

**Affiliations:** 1 Laboratory for Movement Biomechanics, ETH Zürich, 8092 Zürich, Switzerland; 2 Research & Development, Aesculap AG, Am Aesculap Platz, 78532 Tuttlingen, Germany; 3 Department of Orthopaedic and Trauma Surgery, Musculoskeletal University Center Munich (MUM), Campus Grosshadern, LMU Munich, 81377 Munich, Germany; 4 Julius Wolff Institute, Berlin Institute of Health at Charité—Universitätsmedizin Berlin, 13353 Berlin, Germany

**Keywords:** knee contact force, kinematics, EMG, machine learning, CAMS-Knee

## Abstract

This study explores the feasibility of estimating tibiofemoral joint contact forces using deep learning models trained on in vivo biomechanical data. Leveraging the comprehensive CAMS-Knee datasets, we developed and evaluated two machine learning network architectures, a bidirectional Long Short-Term-Memory Network with a Multilayer Perceptron (biLSTM-MLP) and a Temporal Convolutional Network (TCN) model, to predict medial and lateral knee contact forces (KCFs) across various activities of daily living. Using a leave-one-subject-out validation approach, the biLSTM-MLP model achieved root mean square errors (RMSEs) as low as 0.16 body weight (BW) and Pearson correlation coefficients up to 0.98 for the total KCF ($F_{\mathrm{tot}}$) during walking. Although the prediction of individual force components showed slightly lower accuracy, the model consistently demonstrated high predictive accuracy and strong temporal coherence. In contrast to the biLSTM-MLP model, the TCN model showed more variable performance across force components and activities. Leave-one-feature-out analyses underscored the dominant role of lower-limb kinematics and ground reaction forces in driving model accuracy, while EMG features contributed only marginally to the overall predictive performance. Collectively, these findings highlight deep learning as a scalable and reliable alternative to traditional musculoskeletal simulations for personalized knee load estimation, establishing a foundation for future research on larger and more heterogeneous populations.

## 1. Introduction

The human knee is subjected to exceptionally high mechanical loads resulting in substantial knee joint contact forces (KCFs) during daily activities (easily reaching 2–5 times body weight [1,2]). Accurate measurement or estimation of KCFs is clinically important since elevated or imbalanced joint loading is a key mechanical driver in the onset and progression of knee osteoarthritis (KOA) [3]. Monitoring KCFs over time could help identify individuals at risk of developing KOA, support assessment of disease progression, and inform the effectiveness of conservative interventions designed to reduce KCFs [4]. Even after total knee arthroplasty (TKA), there is a critical need for improved methods to assess KCFs and help mitigate biomechanical risk factors affecting implant longevity.

While in vivo measurement of KCFs has been made possible through instrumented knee implants [5], cost and ethical considerations have so far limited their use to small cohorts and specific implant designs, thereby constraining the generalizability of the findings. As a result, developing non-invasive and scalable approaches for estimating KCFs remains critical for optimizing biomechanical outcomes in broader patient populations. To overcome the limitations of in vivo KCF measurement, musculoskeletal (MSK) modelling has emerged as a non-invasive alternative for estimating in vivo joint loading conditions by integrating motion capture data from gait laboratory experiments into biomechanical simulations [6,7]. However, these models generally require extensive subject-specific calibration, and often suffer from limited validation data, leading to a wide range of prediction inaccuracies. Additionally, conventional MSK modelling workflows require substantial human effort and computational resources, making them impractical for large-scale applications or rapid processing in clinical and research settings [2,8]. These limitations highlight the need for more efficient and accessible methods to estimate muscle and joint reaction forces.

In recent years, machine learning (ML) methods have emerged as promising tools in biomechanics, offering scalable, data-driven approaches for predicting joint contact forces. ML models can leverage complex datasets containing kinematic, kinetic, and electromyography (EMG) data to learn nonlinear and complex biomechanical relationships [9,10]. In particular, Long Short-Term Memory (LSTM) neural networks are well-suited for biomechanics applications due to their ability to retain and update sequential information, effectively capturing temporal dependencies in time-series describing human motion. Prior work has demonstrated their ability to accurately predict joint torques and contact forces, especially during cyclic movements with slower velocities [2,10,11,12], underscoring the potential of recurrent architectures for estimating joint kinematics and kinetics. Similarly, Temporal Convolutional Networks (TCNs) offer an alternative sequential modelling approach using dilated causal convolutions to efficiently capture long-range temporal dependencies. Compared to recurrent networks like LSTMs, TCNs can provide several advantages, including enhanced parallelism, improved training stability, and faster convergence [13]. Regarding the aforementioned advantages, TCNs have been successfully applied in biomechanics for motion prediction and joint torque estimation, showing competitive or superior performance compared to other ML models [14].

To date, studies using ML approaches to understand joint biomechanics have generally been limited by the availability of accurate in vivo datasets. For the first time, our study has used the full capacity of the CAMS-Knee datasets [1] to explore the feasibility of using ML models for estimating knee joint loading conditions from non-invasive motion capture data. This comprehensive dataset includes optical motion capture, ground reaction forces (GRFs), EMG recordings, and in vivo KCFs measured from six TKA patients with instrumented implants performing multiple functional tasks [1,5]. We trained and compared a biLSTM network combined with a Multilayer Perceptron (referred to as a biLSTM–MLP [2,10]) with a TCN architecture [13,15] using this data. By comparing the ability of both ML architectures and existing literature to estimate the in vivo measured joint contact forces, this study provides a proof-of-concept assessment of their strengths and limitations for predicting subject-specific knee mechanics during functional activities of daily living.

## 2. Materials and Methods

This study uses the CAMS knee dataset, which contains comprehensive biomechanical data from six patients with instrumented INNEX® FIXUC knee implants [1]. The dataset comprises multimodal recordings, including marker-based motion capture via a VICON system (100 Hz), GRFs collected using fixed and mobile force plates (2000 Hz), and 16-channel surface EMG capturing activation patterns from major lower limb muscles (2000 Hz). The recorded muscles included the gastrocnemius lateralis and medialis, hamstring lateralis and medialis, rectus femoris, tibialis anterior, vastus lateralis, and vastus medialis. Single-plane fluoroscopy data (25 Hz) were also reported, enabling 3D reconstructions of tibiofemoral implant kinematics. In addition, all six components of tibiofemoral contact forces and moments were measured in vivo using instrumented knee implants (up to 100 Hz), along with implantation-specific parameters such as the tibiofemoral varus/valgus angle and posterior tibial slope. Participants performed at least five repetitions of various activities of daily living, including level walking, stair descent, downhill walking on a 10% slope, stand-to-sit and sit-to-stand movements, and squatting, ensuring a diverse representation of joint loading conditions across functional activities. One participant (Subject K3R) did not participate in the downhill walking task [1]. Each activity was one-hot-encoded and included as an additional model input to preserve task-specific variance during training.

To ensure data consistency and optimize input for ML models, a series of preprocessing steps were applied using MATLAB (R2024b, The Mathworks, Inc., Natick, MA, USA). These steps included movement cycle segmentation, filtering, smoothing, alignment, normalization, and coordinate system transformation. EMG signals were normalized to the peak value across all tasks and cycles for each subject individually. It should be noted that, for this study, only EMG signals from the implanted limb were used. GRFs and KCF measurements were normalized by BW. Fluoroscopy-derived kinematic data were processed to extract knee joint angles in three anatomical planes: flexion-extension, abduction-adduction, and internal-external rotation. Moreover, skin-marker trajectories were fed into OpenSim standard inverse kinematic tool to obtain the ankle and hip joint rotation angles during the studied activity cycles [16]. All time-dependent biomechanical data were resampled to a standardized 101-point temporal scale per cycle, enabling temporal alignment across subjects. For static features such as implant alignment parameters or categorical task encodings, a 101-row time series was constructed by repeating the same value across all time steps within each movement cycle, thereby matching the dimensionality of dynamic inputs and ensuring compatibility with sequential model architectures. All input features (Table 1), including kinematics, EMG, and GRF, were z-score normalized. The medial ($F_{\mathrm{med}}$) and lateral ($F_{\mathrm{lat}}$) components of the total KCF were derived from the axial contact force ($F_{z}$) and abduction/adduction moment ($M_{y}$) recorded by the instrumented implants [17].

**Figure 1:**
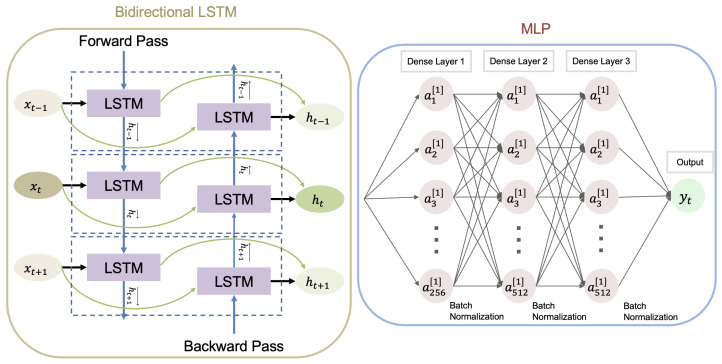
Architecture of the BiLSTM-MLP model used for predicting KCF. Sequential input data were passed through two stacked bidirectional LSTM layers (256 hidden units per direction), producing a 512-dimensional output at each time step. This was followed by a three-layer MLP with dense connections (256 → 512 → 512 units), batch normalization, and ReLU activations. The output layer mapped to three force components: medial ($F_{\mathrm{med}}$), lateral ($F_{\mathrm{lat}}$), and the total KCF ($F_{\mathrm{tot}}$). Adapted from Computers in Biology and Medicine, Vol. 170, Xiang et al. [10], Integrating an LSTM framework for predicting ankle joint biomechanics during gait using inertial sensors, 108016, 2024, with permission from Elsevier.

A BiLSTM-MLP model was implemented, consisting of two stacked bidirectional LSTM layers, each with 256 hidden units and a 40% dropout rate (see Figure 1). The output from both directions of the LSTM was concatenated into a 512-dimensional representation per time step, which was then passed into a multi-layer perceptron (MLP). The MLP architecture included three fully connected layers: the first reduced the dimensionality to 256 units, followed by two hidden layers with 512 units each. Each layer was followed by batch normalization and a Rectified Linear Unit (ReLU) activation function. A final dense output layer with a linear activation function produced three continuous outputs corresponding to the $F_{\mathrm{med}}$, $F_{\mathrm{lat}}$, and $F_{\mathrm{tot}}$ force components. This model was trained using the Adam optimizer with a learning rate of $1\;\times \;10^{-3}$ and RMSE as the loss function. Training proceeded for a maximum of 500 epochs per fold, with early stopping triggered by validation loss and a patience threshold of 50 epochs [10].

**Figure 2:**
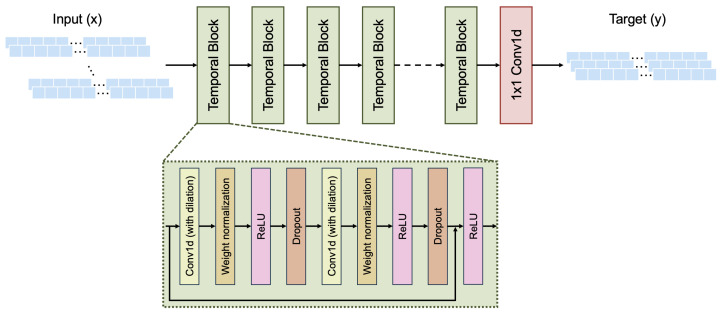
Schematic of the implemented TCN architecture used for predicting KCF. The model includes eight temporal blocks with increasing dilation factors, followed by a 1 × 1 convolution to map to the output force components.

In parallel, a TCN was implemented based on the architecture initially proposed by Bai et al. [13] and further adapted to prosthesis control in a recent study [15]. The network comprised eight temporal convolutional blocks, each consisting of two 1D dilated convolutional layers using a fixed kernel size of 2 and with exponentially increasing dilation factors (1, 2, 4, 8, 16, 32, 64, and 128). To preserve temporal causality and ensure that the output at time step *t* depends only on inputs from time steps ≤ *t*, left-sided causal padding was applied to each convolutional layer. Following each convolution, a cropping operation (commonly referred to as a “chomp”) was applied to remove the extra padding and restore the original sequence length [13]. Each convolutional layer was followed by weight normalization, ReLU activation, and dropout, with residual connections included at the block level to maintain gradient flow. All convolutional layers had 64 output channels. The dropout rate was fixed at 0.0269, consistent with optimized values from prior work [15]. A final 1 × 1 convolutional layer projected the output to three channels representing $F_{\mathrm{med}}$, $F_{\mathrm{lat}}$, and $F_{\mathrm{tot}}$ (see Figure 2). The model was trained using the Adam optimizer with a learning rate of $4.224\;\times \;10^{-5}$, again using RMSE as the loss function and the same early stopping strategy.

For model development and evaluation, a Leave-One-Subject-Out (LOSO) cross-validation strategy was adopted to ensure robust generalization to unseen subjects [18]. In each fold, four subjects were used for training, one for validation, and one for testing. All available activities and movement cycles for each subject were included in their respective data partition. Model training and evaluation were carried out in Python 3.11.2 using the PyTorch 2.3.0 framework within the Visual Studio environment.

Model performance was evaluated quantitatively using RMSE (Equation (1)), normalized RMSE (nRMSE, Equation (2)), and Pearson correlation coefficient (PCC, Equation (3)) to assess both the magnitude of prediction errors and the strength of linear relationships between predicted and in vivo measured forces. In these equations, $y_{i}$ represents the measured force values, ${\hat{y}}_{i}$ the predicted values, *N* the number of data points, $\bar{y}$ and $\bar{\hat{y}}$ the mean values of measured and predicted forces, respectively, and ${\bar{F}}_{\mathrm{peak}}$, the per-cycle peak magnitude of the true force signal. To facilitate comparison across subjects and tasks, RMSE values were normalized by ${\bar{F}}_{\mathrm{peak}}$, resulting in nRMSE (Equation (2)) expressed as a percentage. In addition to numerical metrics, qualitative assessments were conducted by plotting predicted forces alongside the ground truth measurements, depicting mean and standard deviation across trials. This allowed visual evaluation of the model’s ability to capture temporal trends and inter-trial variability [2,9,10,11].

(1) $$\mathrm{RMSE}=\sqrt{\frac{1}{N}\sum_{i=1}^{N}{\left(y_{i}-{\hat{y}}_{i}\right)}^{2}}$$

(2) $$\mathrm{nRMSE}=\frac{\mathrm{RMSE}}{{\bar{F}}_{\mathrm{peak}}}\times 100\%$$

(3) $$\mathrm{PCC}=\frac{\sum_{i=1}^{N}\left(y_{i}-\bar{y}\right)\left({\hat{y}}_{i}-\bar{\hat{y}}\right)}{\sqrt{\sum_{i=1}^{N}{\left(y_{i}-\bar{y}\right)}^{2}}\sqrt{\sum_{i=1}^{N}{\left({\hat{y}}_{i}-\bar{\hat{y}}\right)}^{2}}}$$

**Figure A1:**
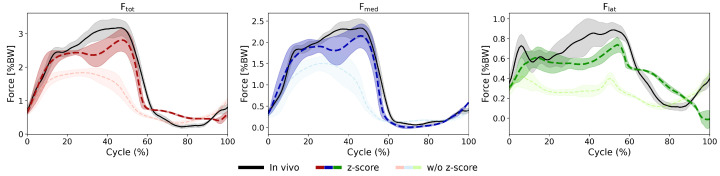
Comparison of prediction performance with and without z-score normalization for an exemplary subject (K1L) during walking. The shaded areas represent ±1 standard deviation across all walking cycles. Predicted force trajectories using normalized inputs (darker red, blue and green) more closely follow the in vivo measured tibiofemoral contact forces (black) than predictions based on raw, unnormalized inputs (brighter red, blue and green). This improvement is evident across all force components ($F_{\mathrm{tot}}$, $F_{\mathrm{med}}$, and $F_{\mathrm{lat}}$), particularly in peak magnitudes and overall pattern alignment, highlighting the importance of input scaling for model accuracy.

To further explore model behaviour and robustness, several additional analyses were conducted. Each model was trained both with and without z-score normalized input features to investigate the effect of input scaling on model performance. However since input normalization significantly improved the model performance (Figure A1, Table A1), results presented in the following section are all obtained using the z-score normalized input features. Moreover, in addition to the LOSO framework, a Leave-One-Trial-Out (LOTO) cross-validation was performed to evaluate intra-subject generalization across individual repetitions. Finally, feature importance was analysed by systematically removing one input feature at a time, namely EMG signals, kinematics, or GRFs, and observing the resulting change in prediction accuracy. This leave-one-feature-out (LOFO) approach provided insights into the relative contribution of different input modalities to overall model performance.

## 3. Results

**Figure 3:**
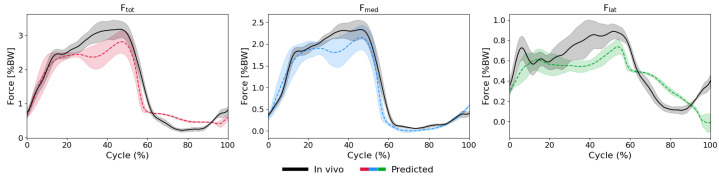
Predicted (dashed lines) versus in vivo measured (solid black lines) KCFs during level walking for an exemplary subject. The shaded areas represent ±1 standard deviation across all walking cycles. The three force components shown are $F_{\mathrm{tot}}$ (**left**), $F_{\mathrm{med}}$ (**middle**), and $F_{\mathrm{lat}}$ (**right**) KCFs.

**Figure A2:**
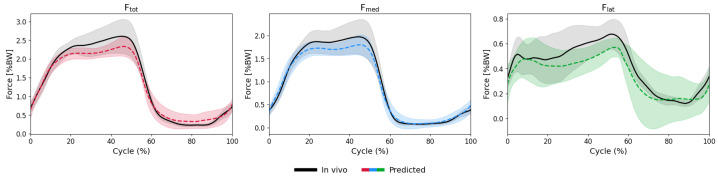
**Walking**: Average predicted (red, blue, green) and in vivo (black) KCFs across all subjects for level walking. $F_{\mathrm{tot}}$ and $F_{\mathrm{med}}$ show strong agreement and low variability, $F_{\mathrm{lat}}$ displays slightly higher inter-subject variation. The model captures the general loading pattern with some general underestimation.

**Figure A3:**
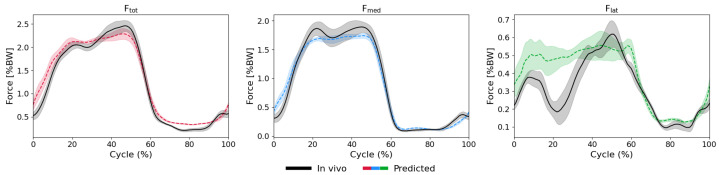
**Walking—K8L**: Average predicted (red, blue, green) and in vivo (black) KCFs across all trials of level walking.

**Figure A4:**
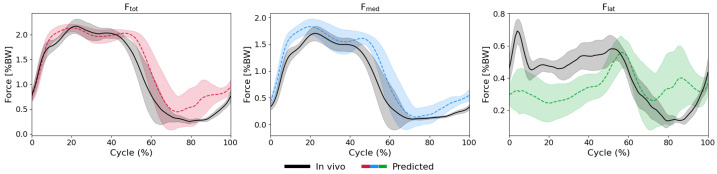
**Walking—K3R**: Average predicted (red, blue, green) and in vivo (black) KCFs across all trials of level walking.

**Figure A5:**
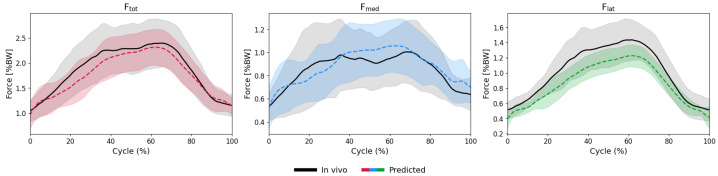
**Squat**: Average predicted (red, blue, green) and in vivo (black) KCFs across all subjects for squatting. Greater variability is observed compared to level walking, particularly in $F_{\mathrm{med}}$.

**Figure A6:**
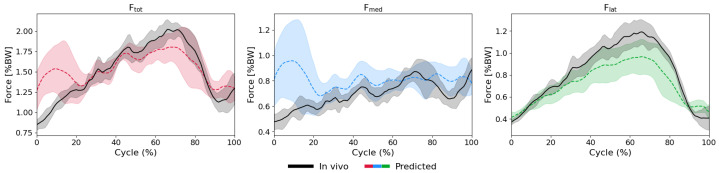
**Squat—K7L**: Average predicted (red, blue, green) and in vivo (black) KCFs across all trials of squatting.

**Figure A7:**
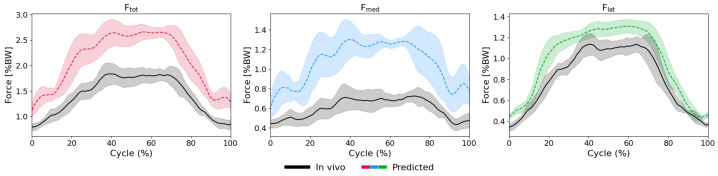
**Squat—K3R**: Average predicted (red, blue, green) and in vivo (black) KCFs across all trials of squatting.

The biLSTM-MLP model demonstrated strong performance in predicting medial ($F_{\mathrm{med}}$), lateral ($F_{\mathrm{lat}}$), and total ($F_{\mathrm{tot}}$) KCFs with good accuracy across all activities and subjects. LOSO validation revealed relatively low nRMSEs during walking across subjects, with peak nRMSE values of 11.9% relative to the true peak force (PCC: 0.97) for $F_{\mathrm{tot}}$, 11.9% (0.98) for $F_{\mathrm{med}}$, and 27.8% (0.66) for $F_{\mathrm{lat}}$. (Figure 3 and Figure A2, Figure A3 and Figure A4) However, for the squatting activity, the errors were relatively higher: 23.4% (0.84) for $F_{\mathrm{tot}}$, 34.9% (0.61) $F_{\mathrm{med}}$, and 17.8% (0.92) for $F_{\mathrm{lat}}$ (Table 2, Figure A5, Figure A6 and Figure A7).

**Figure 4:**
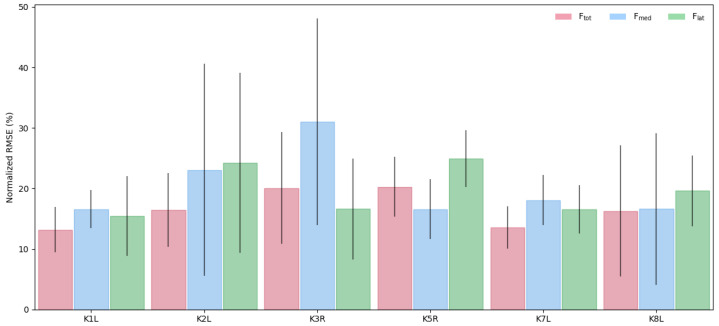
Average nRMSE (%) values of the predicted force components ($F_{\mathrm{tot}}$, $F_{\mathrm{med}}$, $F_{\mathrm{lat}}$) across all tasks performed by the subjects. Error bars represent ±1 standard deviation.

**Figure A8:**
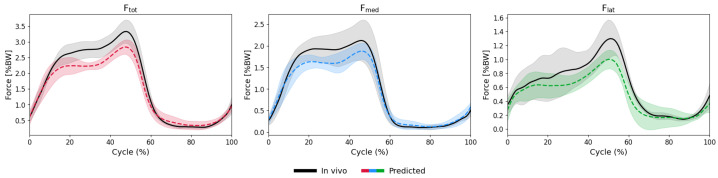
**Downhill walking**: Average predicted (red, blue, green) and in vivo (black) KCFs across all subjects for downhill walking. Across all force components the forces where underestimated and showed lower variability.

**Figure A9:**
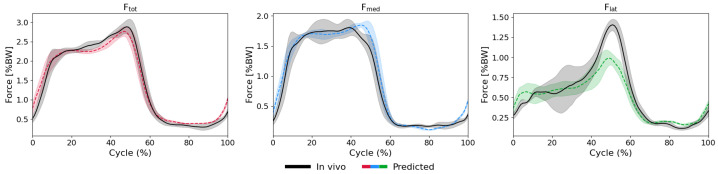
**Downhill walking—K8L**: Average predicted (red, blue, green) and in vivo (black) KCFs across all trials of downhill walking.

**Figure A10:**
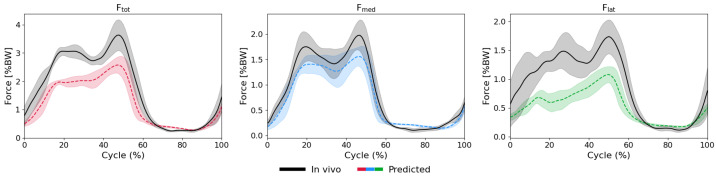
**Downhill walking—K5R**: Average predicted (red, blue, green) and in vivo (black) KCFs across all trials of downhill walking.

**Figure A11:**
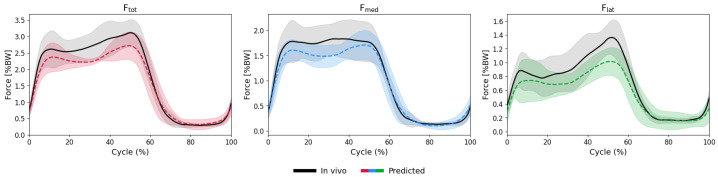
**Stairs descent**: Average predicted (red, blue, green) and in vivo (black) KCFs across all subjects for stair descending. The model captures the general loading pattern with some general underestimation.

**Figure A12:**
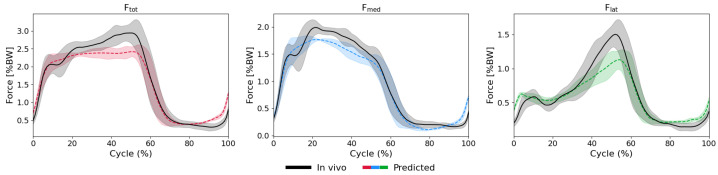
**Stairs descent—K8L**: Average predicted (red, blue, green) and in vivo (black) KCFs across all trials of stair descending.

**Figure A13:**
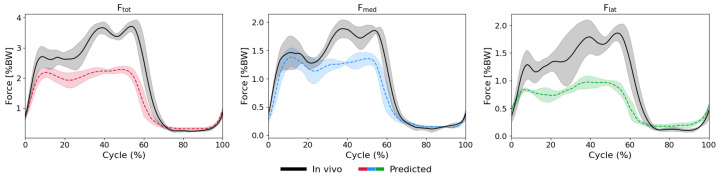
**Stairs descent—K5R**: Average predicted (red, blue, green) and in vivo (black) KCFs across all trials of stair descending.

**Figure A14:**
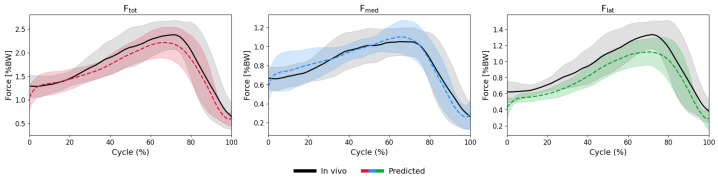
**Sit down**: Average predicted (red, blue, green) and in vivo (black) KCFs across all subjects for sitting down. Overall trends align well, though with smaller predicted variability.

**Figure A15:**
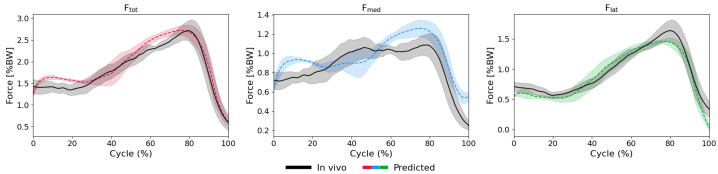
**Sit down—K1L**: Average predicted (red, blue, green) and in vivo (black) KCFs across all trials of sitting down.

**Figure A16:**
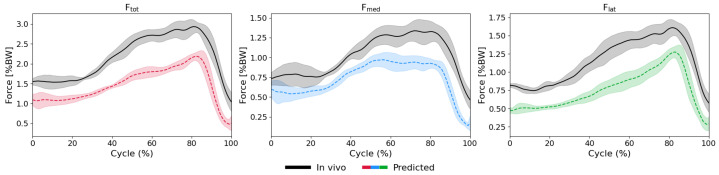
**Sit down—K8L**: Average predicted (red, blue, green) and in vivo (black) KCFs across all trials of sitting down.

**Figure A17:**
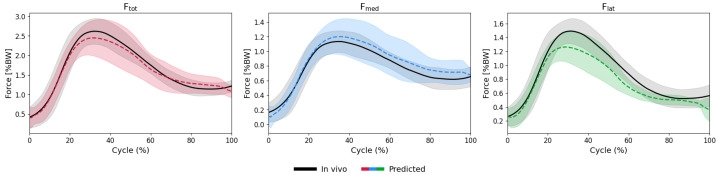
**Stand up**: Average predicted (red, blue, green) and in vivo (black) KCFs across all subjects for standing up. The predictions align well with the in vivo force profiles, particularly in the early loading phase.

**Figure A18:**
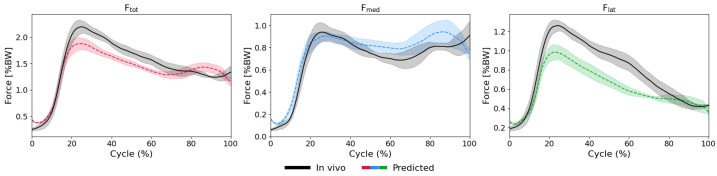
**Stand up—K7L**: Average predicted (red, blue, green) and in vivo (black) KCFs across all trials of standing up.

**Figure A19:**
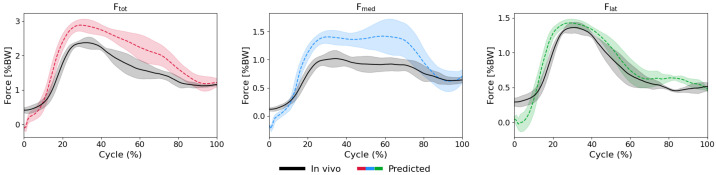
**Stand up—K2L**: Average predicted (red, blue, green) and in vivo (black) KCFs across all trials of standing up.

**Figure A20:**
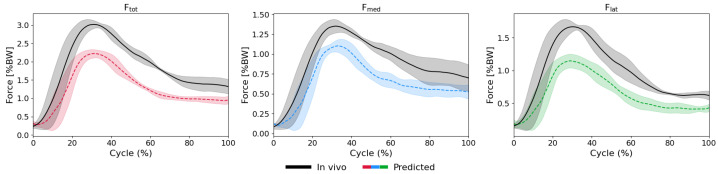
**Stand up—K5R**: Average predicted (red, blue, green) and in vivo (black) KCFs across all trials of standing up.

Importantly, the timing of the peaks and the overall patterns of the measured force components were accurately predicted by the model (Figure 3, Figure A2, Figure A3, Figure A4, Figure A5, Figure A6, Figure A7, Figure A8, Figure A9, Figure A10, Figure A11, Figure A12, Figure A13, Figure A14, Figure A15, Figure A16, Figure A17, Figure A18, Figure A19 and Figure A20). However, when comparing predictive capacity of the model across the force components, the model generally exhibited the strongest performance in predicting $F_{\mathrm{tot}}$, with the lowest nRMSE values (typically below 20%, Table 2) and consistently high PCCs (mostly ≥ 0.9, Table 2). $F_{\mathrm{med}}$ and $F_{\mathrm{lat}}$ predictions showed similar trends as those for $F_{\mathrm{tot}}$ but with larger variability and some clear outliers reaching nRMSEs of up to 60%, specifically for the squat task (Figure 4 and Figure A7).

**Figure 5:**
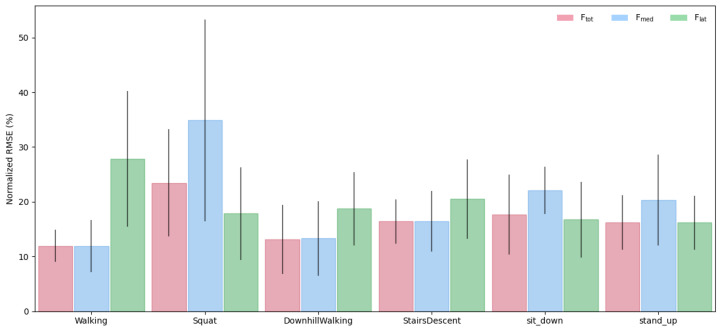
Average nRMSE (%) across subjects for each activity and force component ($F_{\mathrm{tot}}$, $F_{\mathrm{med}}$, $F_{\mathrm{lat}}$). Error bars indicate ±1 standard deviation. The model yielded the best predictive performance for $F_{\mathrm{tot}}$ and $F_{\mathrm{med}}$ during walking, while the $F_{\mathrm{med}}$ estimates for squat activity showed the largest prediction errors.

Comparing the model performance across tasks indicates a relatively consistent performance, with most activities yielding nRMSE values between 12 and 20% (Figure 5). The model achieved the best overall performance for $F_{\mathrm{tot}}$ and $F_{\mathrm{med}}$ during walking task; however, predictions for the $F_{\mathrm{lat}}$ component were significantly larger (Figure 5 and Figure A3). In contrast, the model demonstrated the weakest performance for the squat activity, especially for $F_{\mathrm{med}}$, with prediction errors of up to 50% (Figure 5 and Figure A7).

### 3.1. LSTM Model: Intra-Subject Predictions (LOTO)

**Figure 6:**
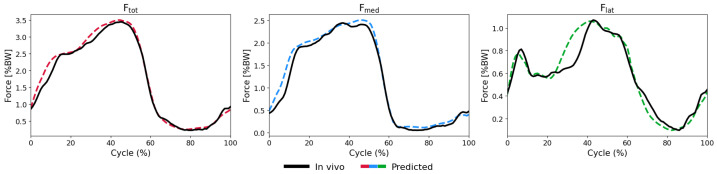
Example of intra-subject prediction of the force components using the LOTO approach for walking trials performed by an exemplary subject. Predicted force trajectories (red, blue, green) closely match the in vivo measured tibiofemoral contact forces (black solid lines) for all force components ($F_{\mathrm{tot}}$, $F_{\mathrm{med}}$, and $F_{\mathrm{lat}}$).

LOTO evaluation confirmed that the biLSTM-MLP model can generalize well to unseen repetitions of the same activity within each individual subject. With 6.5% ± 4.4% (PCC: 0.98 ± 0.1) for $F_{\mathrm{tot}}$, 8.6% ± 5.9% (0.93 ± 0.2) for $F_{\mathrm{med}}$, and 9.0% ± 5.5% (0.96 ± 0.1) for $F_{\mathrm{lat}}$, nRMSE values were considerably lower compared to the LOSO approach. Here, temporal patterns were reliably preserved across repetitions, as demonstrated by high PCCs and closely overlapping predicted and ground-truth trajectories (see Figure 6).

### 3.2. LSTM Model: Feature Importance Analysis

**Figure 7:**
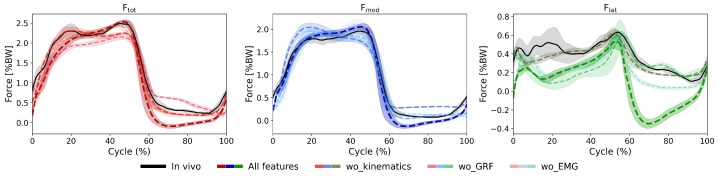
Effects of input feature exclusion on force predictions for walking cycles performed by an exemplary subject. The shaded areas represent ±1 standard deviation across all walking cycles. The predicted KCF trajectories using all input features are compared against predictions without input kinematics, GRF, or EMG. For all force components ($F_{\mathrm{tot}}$, $F_{\mathrm{med}}$, $F_{\mathrm{lat}}$), excluding input kinematics led to the largest increase in prediction errors, demonstrating inaccurate force patterns and magnitudes.

The LOFO analysis revealed differences in the contribution of individual input modalities to model performance. Overall, lower-limb kinematics emerged as the most critical input, particularly for predicting $F_{\mathrm{med}}$ and $F_{\mathrm{lat}}$ (Figure 7, Table 3). For all studied activities, excluding kinematic inputs consistently led to notable increases in nRMSE (e.g., for walking, $F_{\mathrm{tot}}$ errors increased from 11.9% to 13.3%, and for the squat, $F_{\mathrm{tot}}$ errors rose from 23.4% to 31.0%), with considerable decreases in correlation coefficients. Contrary, the implant alignment parameters had only a negligible impact on the model prediction performance.

**Figure 8:**
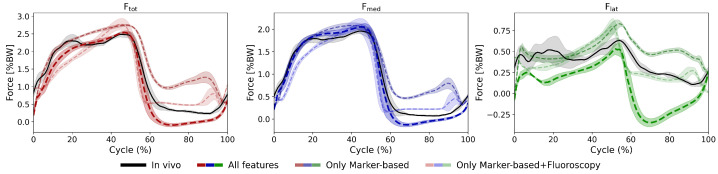
Impact of Kinematic Input Feature Selection on Force Predictions During Walking Cycles of an Exemplary Subject. The shaded areas represent ±1 standard deviation across all walking cycles. Predicted KCF trajectories using all input features are compared with predictions based on marker-based only and marker-based combined with fluoroscopic kinematic inputs. For all force components ($F_{\mathrm{tot}}$, $F_{\mathrm{med}}$, $F_{\mathrm{lat}}$), using only skin marker kinematics as inputs resulted in a substantial increase in prediction errors, affecting both the force patterns and their magnitudes.

Predictions based solely on marker-based kinematic inputs were generally less accurate compared to those using all available features (Table 3, Figure 8). Here, including fluoroscopic knee kinematics alongside marker-based kinematics improved accuracy, with gains ranging from 0.5% to 8.3% across force components.

Removing GRFs reduced the model performance, though with inconsistent impact magnitudes (e.g., nRMSE increased from 11.9% to 14.4% for walking, and from 23.4% to 24.7% for squatting). Interestingly, excluding EMG data had only a minor impact on the overall predictive performance. For instance, removing EMG resulted in negligible changes in nRMSE of force components during walking (12.1% $F_{\mathrm{tot}}$ error without EMG compared to 11.9% error for all inputs). Similarly, when removing the input EMG signals, error in $F_{\mathrm{tot}}$ predictions for the squat activity was only slightly increased (from 23.4% to 25.1%).

### 3.3. LSTM vs. TCN

**Figure 9:**
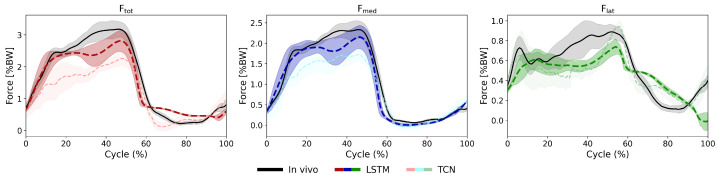
Comparison of predicted KCF components between LSTM and TCN models against in vivo measurements (black lines) for an exemplary subject during walking. The shaded areas represent ±1 standard deviation across all walking cycles. The LSTM model predictions more closely align with the in vivo data, especially in peak magnitudes and temporal patterns. The TCN model shows a tendency to underpredict force peaks and smooth out dynamic features, indicating reduced temporal precision compared to the LSTM.

The influence of network architecture on prediction accuracy was assessed by a systematic comparison between the biLSTM-MLP and TCN model performance. In general, the LSTM consistently outperformed the TCN model, as indicated by smaller nRMSEs and larger PCCs. For instance, LSTM achieved an average nRMSE of 11.9% with a PCC of 0.97 for the $F_{\mathrm{tot}}$ during walking, whereas the TCN predictions showed a larger error (17.6%) and a lower PCC (0.95) (Figure 9). Similar trends were observed across all other activities, with LSTM yielding lower RMSEs and higher correlation coefficients, highlighting its more robust temporal alignment and prediction fidelity.

## 4. Discussion

### 4.1. Model Performance and Validation

This study investigated the feasibility of estimating KCF using ML methods, based on data from the CAMS-Knee dataset, the most comprehensive in vivo knee biomechanics dataset currently available. Two ML models were developed and trained on biomechanical data, including kinematics, GRF, and EMG, to predict subject-specific medial, lateral, and total KCF across various functional tasks. The results provide a proof-of-concept demonstration that LSTM networks can accurately estimate KCFs from non-invasive biomechanical data. This approach provides a scalable, cost-efficient, and non-invasive tool for estimation of the KCF during functional activities, with potential applications in personalized MSK assessment and rehabilitation optimization.

Despite task- and subject-specific variability, the ML models were able to predict KCFs with good accuracy across a range of activities, with the biLSTM-MLP outperforming the TCN. These findings confirm that ML models, when trained on reliable biomechanical input data, can capture subject-specific force patterns and produce temporally coherent predictions. Overall, the predicted force trajectories using ML models showed strong temporal agreement with the in vivo measurements (PCCs mostly ≥ 0.6, Table 2), particularly for walking (PCCs ~ 0.93, Table 2), highlighting the model’s robustness in capturing task-specific neuromechanical patterns. The lower prediction performance for KCFs during squatting (Figure 5 and Table 2) suggests that tasks involving distinct neuromechanical dynamics may challenge the model’s ability to generalize. Squatting typically requires substantial co-contraction of the knee extensors and flexors to maintain postural stability. It is well established that, despite exhibiting similar kinematics, individuals may adopt different stabilization strategies depending on factors such as pain, neuromuscular control, or soft-tissue stiffness [19]. Future studies could benefit from collecting datasets that, in addition to the parameters captured by the CAMS-Knee project, include measures of pain, joint stability, and muscle recruitment patterns, ideally across larger cohorts. ML models trained on such enriched data may generalize more reliably across multiple tasks and subjects.

### 4.2. Comparison with Existing Approaches

When compared to recent studies using different ML-based approaches, our biLSTM-MLP model demonstrated notable improvements in both magnitude and temporal accuracy of KCF predictions. In particular, Bennett et al. [2] reported RMSEs ranging 0.23–0.59 BW (0.20 < $R^{2}$ < 0.88) for KCF during walking. Here, our model achieved considerably smaller RMSEs (0.12–0.21 BW) and superior correlation metrics (0.36 < $R^{2}$ < 0.97), when tested on the same subjects. Similarly, relative to earlier modelling approaches [20,21], our model achieved better agreement with experimental data. The superior outcomes from our ML models compared to previously developed models may be attributed to several key factors: the use of comprehensive in vivo data (CAMS-Knee), a larger and more diverse training set, and the integration of multimodal input features (kinematics, GRF, EMG, implantation-specific parameters). Furthermore, the use of a biLSTM-MLP architecture in the current study allowed for effective temporal modelling of biomechanical signals, likely contributing to improved capture of dynamic loading patterns across functional tasks. However, the predicted force trajectories using TCN showed slightly lower temporal agreement with in vivo measurements and larger variability across force components compared to biLSTM (Table 4). One possible reason for the TCN’s lower stability and performance may originate from its reliance on convolutional filters that capture local temporal patterns but struggle to model longer-range dependencies as effectively as biLSTMs, which are designed to retain information over extended sequences. Additionally, the TCN’s fixed receptive field might limit its adaptability across highly variable and complex biomechanical tasks, where force dynamics can differ substantially between individuals.

### 4.3. Feature Importance and Input Contributions

Our findings revealed that lower-limb kinematics and GRF data were the most influential input features for KCF prediction (Figure 7). The LOFO analysis (Table 3) showed that different input modalities contribute differently: Our results indicate that GRF primarily influenced $F_{\mathrm{tot}}$ prediction accuracy, with its removal increasing errors more substantially. This can be justified by multibody dynamic principles, since force equilibrium suggest larger overall knee joint reaction forces in response to elevated GRFs. Our findings also show that fluoroscopic joint kinematics were particularly critical for predicting medial-lateral force distribution ($F_{\mathrm{med}}$ and $F_{\mathrm{lat}}$). Biomechanically, joint alignment and orientation directly determine how loads are distributed across knee compartments. Here, fluoroscopic kinematics (which provide more accurate joint angles than marker-based measurements) can improve prediction accuracy for these compartment-specific forces (Table 3). Detailed analysis of different kinematic input combinations (Appendix A.2 Table A2) shows that marker-based motion capture alone can provide acceptable performance, though strong correlations between kinematic features make it difficult to isolate the specific contribution of each modality. Notably, even when using only skin-marker-based kinematics, the model was able to predict KCFs reasonably well, with an average error of 24.4% (Table 3). The unexpected low contribution of EMG signals towards the overall model performance may be due to noise, electromechanical delay, or crosstalk inherent in surface EMG. It is also known that EMG signals are often strongly correlated with other biomechanical variables, e.g., joint angles. The highly correlated input features can lead LSTM models to overfit or struggle to learn distinct patterns, as redundancy may dilute the model’s attention to the most informative inputs [22,23]. Our findings suggest that, under certain conditions, simplified input sets may still yield acceptable accuracy. Future work may use composite metrics such as the co-contraction index (CCI) that reduces the input EMG data dimensionality. Summarizing multiple muscle EMG in CCI can also enhance physiological interpretability, as a higher co-contraction is often associated with increased joint loading [24].

### 4.4. Clinical Applications and Future Directions

The ability to predict KCFs non-invasively and rapidly holds substantial promise for clinical applications. Efficient processing of complete movement cycles could enable timely biofeedback for rehabilitation or gait retraining, helping patients minimize harmful joint loading and potentially reduce pain. Previous work has shown that immediate feedback during gait retraining can effectively reduce surrogate loading parameters like the knee adduction moment, improving symptoms and function in individuals with KOA [25,26]. The LOTO evaluation of our ML model demonstrated its ability to generalize to unseen trials performed by the same subject, with consistent performance across different activities, highlighting the approach’s suitability for personalized monitoring applications. The substantially lower prediction errors in LOTO compared to LOSO validation reflect the model’s capacity to learn subject-specific biomechanical patterns, whereas LOSO provides a more conservative estimate of the model’s ability to generalize to completely unseen individuals, a key requirement for clinical deployment without subject-specific calibration. Nonetheless, applying ML models trained on individuals with intact neuromuscular function to populations with altered motor control (e.g., due to cerebral palsy, Parkinson’s disease, or stroke) requires caution, as greater variability and atypical movement patterns may limit generalizability [27,28,29]. Our current biLSTM-MLP model shows strong predictive performance; however, its clinical deployment may require broader training and validation across diverse patient populations and movement contexts. Future work should focus on expanding the training and validation datasets preferably with more subjects equipped with instrumented implants. Although the acquisition of such in vivo data remains rare due to technical and ethical constraints [5], even small additions, particularly from individuals with diverse anthropometric characteristics and clinical backgrounds, could substantially improve model generalizability. A complementary strategy is to leverage the physics-informed nature of MSK modelling, for example as a pre-training prior or a data-augmentation source [30]. In this scheme, the network first learns general biomechanical relationships under idealized, physics-consistent conditions and is then fine-tuned on smaller, more variable in vivo datasets, improving robustness and sample efficiency [31]. While the prediction of absolute force magnitudes using the current ML model was limited in accuracy, the model reliably captured the shape and timing of the force curves. This temporal fidelity remains valuable for applications focused on movement quality, trend tracking, or biofeedback.

### 4.5. Limitations

Several limitations of the current study may need to be considered while interpreting the findings reported. First, the CAMS-Knee datasets used in this study included only six subjects. However, despite the limited number of subjects (*n* = 6), the dataset still captured meaningful inter-subject variability. The included individuals differed substantially in age (66–79 years), body mass (67–101 kg), and height (165–175 cm), as well as surgical parameters such as tibiofemoral alignment (1.0–6.5° varus) and posterior slope (5–11°) [1,19]. Moreover, the studied activities capture a broad spectrum of functional movement types, from quasi-static (e.g., squatting) to dynamic tasks with impact (e.g., stair descent), thus covering a wide range of joint loading conditions. While further data would improve robustness, this cohort already spans a wide clinical and biomechanical spectrum relevant for joint loading analysis. Another limitation lies in the small number of ML model architectures tested and the lack of systematic hyperparameter optimization. We only compared two model architectures (biLSTM-MLP and TCN) and did not perform comprehensive hyperparameter tuning or architecture search for either model. Instead, we adopted configurations that were previously validated in biomechanical applications [10,13,15]. While this approach allowed for a fair comparison between established architectures, task-specific optimization could potentially improve model performance. For instance, the TCN parameters optimized for prosthesis control [15] may not be optimal for knee force prediction, potentially affecting the performance comparison between the two networks. Future studies could explore alternative or hybrid ML models with task-specific hyperparameter optimization, potentially incorporating physics-informed layers or uncertainty quantification with task-specific optimization. Finally, our ML models were trained on individuals with TKA but with intact neuromuscular function. Caution is warranted when applying these models to populations with altered motor control (e.g., due to cerebral palsy or Parkinson’s disease), as increased variability and atypical movement patterns, unseen during training, may limit model generalizability. Similarly, direct application to individuals without TKA, such as healthy subjects or those with early-stage knee osteoarthritis, requires careful consideration, as these populations may exhibit different joint mechanics, soft tissue constraints, and loading patterns compared to post-TKA patients. However, currently, obtaining ground truth in vivo force measurements in non-implanted populations is not ethically or practically feasible, presenting a fundamental challenge for model validation beyond TKA cohorts.

## 5. Conclusions

This study provides a proof-of-concept demonstration that deep learning models can estimate tibiofemoral joint contact forces from non-invasive biomechanical data. Using the comprehensive CAMS-Knee dataset, we trained and compared two network architectures (biLSTM-MLP and TCN) and showed that the biLSTM-MLP achieved high predictive accuracy across multiple activities of daily living. These findings highlight the potential of deep learning as a scalable, cost-efficient, and non-invasive alternative to MSK simulations for assessing knee loading. While the present work was conducted on a modest cohort, the results establish a foundation for future research on larger and more diverse populations, with applications ranging from rehabilitation and clinical monitoring to personalized MSK modelling and performance assessment.

## Figures and Tables

**Table 1:** Input features for the ML models.

Implantation Features	EMG	Skin-Marker Kinematics	Fluoroscopic Kinematics	GRF	Tasks
Frontal plane limb alignment	Gastrocnemius lateralis	Knee flexion	Knee flexion	Superior force	Walking
Posterior tibial slope	Gastrocnemius medialis	Hip flexion	Knee abduction	Anterior force	Squat
	Hamstring lateralis	Hip adduction	Knee rotation	Medial force	Downhill Walking
	Hamstring medialis	Hip rotation		Free torque	Stairs Descent
	Rectus femoris	Ankle flexion			Sit Down
	Tibialis anterior				Stand Up
	Vastus lateralis				
	Vastus medialis				

**Table 2:** Performance metrics of the biLSTM-MLP model for different subjects and activities. Each cell contains the nRMSE (%), followed by the PCC in parentheses, both reported as the mean across all cycles performed by the subject. The rightmost column summarizes the average performance across subjects for each task. For each subject, predictions with the largest and smallest errors are highlighted in bold. Subject codes indicate individual participants (K1–K8) and implant side (L = left, R = right).

		K1L	K2L	K3R	K5R	K7L	K8L	Average
**Walking**	$F_{\mathrm{tot}}$	12.3% (0.96)	12.3% (0.99)	**14.4% (0.93)**	**11.6% (0.99)**	14.3% (0.97)	6.7% (0.99)	**11.9% (0.97)**
	$F_{\mathrm{med}}$	11.4% (0.97)	8.7% (0.99)	17.0% (0.96)	10.1% (0.98)	17.9% (0.99)	6.3% (0.99)	11.9% (0.98)
	$F_{\mathrm{lat}}$	21.3% (0.74)	51.4% (0.73)	30.9% (0.23)	19.2% (0.85)	23.8% (0.64)	20.1% (0.79)	27.8% (0.66)
**Squat**	$F_{\mathrm{tot}}$	14.2% (0.71)	20.2% (0.89)	**36.3% (0.97)**	**25.4% (0.89)**	12.3% (0.67)	**32.2% (0.93)**	**23.4% (0.84)**
	$F_{\mathrm{med}}$	19.7% (0.00)	51.8% (0.75)	60.5% (0.87)	18.3% (0.89)	20.4% (0.38)	38.4% (0.74)	34.9% (0.61)
	$F_{\mathrm{lat}}$	11.3% (0.92)	11.6% (0.88)	15.5% (0.97)	31.5% (0.85)	12.5% (0.92)	24.6% (0.94)	17.8% (0.92)
**Downhill Walking**	$F_{\mathrm{tot}}$	**17.0% (0.94)**	**7.3% (0.99)**	NA	18.4% (0.98)	**17.4% (0.99)**	**5.6% (0.99)**	13.1% (0.98)
	$F_{\mathrm{med}}$	19.0% (0.93)	7.0% (0.99)	NA	11.4% (0.97)	21.7% (0.98)	7.5% (0.98)	13.3% (0.97)
	$F_{\mathrm{lat}}$	15.5% (0.88)	24.3% (0.88)	NA	27.1% (0.92)	14.1% (0.88)	12.6% (0.93)	18.7% (0.90)
**Stairs** **Descent**	$F_{\mathrm{tot}}$	16.8% (0.92)	15.4% (0.98)	16.4% (0.97)	22.5% (0.95)	17.1% (0.98)	10.1% (0.97)	16.4% (0.96)
	$F_{\mathrm{med}}$	17.6% (0.88)	12.4% (0.98)	24.2% (0.94)	17.7% (0.95)	18.5% (0.97)	8.4% (0.98)	16.5% (0.95)
	$F_{\mathrm{lat}}$	25.0% (0.86)	27.9% (0.93)	13.7% (0.87)	27.7% (0.92)	16.0% (0.93)	12.6% (0.93)	20.5% (0.91)
**Sit Down**	$F_{\mathrm{tot}}$	**7.3% (0.96)**	**23.1% (0.78)**	16.6% (0.96)	23.0% (0.93)	11.0% (0.83)	24.7% (0.94)	17.6% (0.90)
	$F_{\mathrm{med}}$	15.4% (0.79)	24.8% (0.79)	27.7% (0.93)	22.5% (0.93)	19.7% (0.62)	22.6% (0.90)	22.1% (0.83)
	$F_{\mathrm{lat}}$	8.3% (0.96)	17.8% (0.79)	9.9% (0.94)	22.9% (0.92)	16.2% (0.93)	25.4% (0.94)	16.8% (0.91)
**Stand Up**	$F_{\mathrm{tot}}$	11.5% (0.94)	20.6% (0.96)	16.6% (0.94)	20.7% (0.98)	**9.1% (0.96)**	18.6% (0.98)	16.2% (0.96)
	$F_{\mathrm{med}}$	16.3% (0.93)	33.5% (0.96)	25.7% (0.94)	19.5% (0.97)	10.3% (0.94)	16.4% (0.89)	20.3% (0.94)
	$F_{\mathrm{lat}}$	11.2% (0.93)	12.6% (0.94)	12.9% (0.94)	21.3% (0.98)	16.6% (0.95)	22.4% (0.97)	16.2% (0.95)

**Table 3:** LOFO validation results for different activities and force components. Reported values represent nRMSE (%) and PCC in parentheses, averaged across all cycles and subjects. “With all inputs” reflects the baseline model using all input features. Subsequent columns show performance when using only selected kinematic features as inputs or after excluding one input modality (kinematics, GRF, or EMG). For each task the predictions for $F_{\mathrm{tot}}$ with the largest and smallest errors are highlighted in bold.

		With All Inputs	With Only Marker-Based Kinematics	With Only Marker-Basedand Fluoroscopic Kinematics	Without Kinematics	Without GRF	Without EMG
**Walking**	$F_{\mathrm{tot}}$	**11.9% (0.97)**	**19.5%** **(0.90)**	16.5% (0.93)	13.3% (0.96)	14.4% (0.92)	12.1% (0.97)
	$F_{\mathrm{med}}$	11.9% (0.98)	18.7% (0.91)	17.8% (0.94)	15.1% (0.96)	15.4% (0.93)	14.5% (0.97)
	$F_{\mathrm{lat}}$	27.8% (0.66)	23.9% (0.69)	25.5% (0.74)	20.7% (0.72)	24.0% (0.65)	24.0% (0.78)
**Squat**	$F_{\mathrm{tot}}$	**23.4% (0.84)**	26.4% (0.72)	26.9% (0.62)	**31.0% (0.81)**	24.7% (0.85)	25.1% (0.84)
	$F_{\mathrm{med}}$	34.9% (0.61)	40.0% (0.54)	31.7% (0.32)	37.2% (0.63)	31.6% (0.59)	38.5% (0.66)
	$F_{\mathrm{lat}}$	17.8% (0.92)	23.4% (0.74)	26.5% (0.72)	30.5% (0.89)	24.8% (0.88)	20.7% (0.88)
**Downhill** **Walking**	$F_{\mathrm{tot}}$	13.1% (0.98)	**19.2% (0.91)**	17.9% (0.92)	**13.0% (0.97)**	15.9% (0.95)	13.5% (0.96)
	$F_{\mathrm{med}}$	13.3% (0.97)	20.7% (0.88)	20.0% (0.91)	16.6% (0.96)	17.0% (0.93)	15.0% (0.96)
	$F_{\mathrm{lat}}$	18.7% (0.90)	19.2% (0.87)	20.2% (0.86)	19.1% (0.82)	20.2% (0.79)	17.3% (0.89)
**Stairs** **Descent**	$F_{\mathrm{tot}}$	**16.4% (0.96)**	**22.5% (0.91)**	20.9% (0.92)	17.6% (0.96)	19.1% (0.95)	17.1% (0.96)
	$F_{\mathrm{med}}$	16.5% (0.95)	22.0% (0.88)	21.5% (0.89)	18.7% (0.94)	20.0% (0.93)	18.2% (0.95)
	$F_{\mathrm{lat}}$	20.5% (0.91)	22.3% (0.86)	22.8% (0.88)	21.1% (0.84)	20.7% (0.82)	20.1% (0.90)
**Sit Down**	$F_{\mathrm{tot}}$	**17.6% (0.90)**	26.3% (0.74)	23.0% (0.75)	**28.2% (0.87)**	19.9% (0.85)	19.8% (0.92)
	$F_{\mathrm{med}}$	22.1% (0.83)	29.1% (0.70)	23.6% (0.66)	27.0% (0.84)	22.5% (0.76)	23.4% (0.87)
	$F_{\mathrm{lat}}$	16.8% (0.91)	25.7% (0.68)	26.6% (0.74)	30.5% (0.87)	19.9% (0.85)	21.4% (0.88)
**Stand Up**	$F_{\mathrm{tot}}$	**16.2% (0.96)**	**26.3% (0.79)**	25.0% (0.75)	20.3% (0.90)	17.6% (0.92)	16.2% (0.96)
	$F_{\mathrm{med}}$	20.3% (0.94)	27.4% (0.78)	26.8% (0.70)	22.3% (0.86)	19.6% (0.86)	20.4% (0.92)
	$F_{\mathrm{lat}}$	16.2% (0.95)	26.4% (0.67)	27.6% (0.61)	23.3% (0.87)	20.7% (0.87)	19.3% (0.94)

**Table 4:** Comparison of average model performance between the biLSTM-MLP and TCN model architectures. Each cell contains the nRMSE (%) followed by the PCC in parentheses, averaged across all cycles and subjects. The biLSTM-MLP consistently outperformed the TCN across most tasks and force components, particularly for walking and squatting.

		biLSTM-MLP	TCN
**Walking**	$F_{\mathrm{tot}}$	11.9% (0.97)	17.6% (0.95)
	$F_{\mathrm{med}}$	11.9% (0.98)	17.2% (0.97)
	$F_{\mathrm{lat}}$	27.8% (0.66)	25.8% (0.67)
**Squat**	$F_{\mathrm{tot}}$	23.4% (0.84)	28.8% (0.75)
	$F_{\mathrm{med}}$	34.9% (0.61)	34.4% (0.65)
	$F_{\mathrm{lat}}$	17.8% (0.92)	25.2% (0.74)
**Downhill** **Walking**	$F_{\mathrm{tot}}$	13.1% (0.98)	18.4% (0.96)
	$F_{\mathrm{med}}$	13.3% (0.97)	18.8% (0.96)
	$F_{\mathrm{lat}}$	18.7% (0.90)	19.8% (0.86)
**Stairs** **Descent**	$F_{\mathrm{tot}}$	16.4% (0.96)	20.5% (0.96)
	$F_{\mathrm{med}}$	16.5% (0.95)	20.8% (0.94)
	$F_{\mathrm{lat}}$	20.5% (0.91)	21.7% (0.86)
**Sit Down**	$F_{\mathrm{tot}}$	17.6% (0.90)	24.0% (0.87)
	$F_{\mathrm{med}}$	22.1% (0.83)	26.3% (0.79)
	$F_{\mathrm{lat}}$	16.8% (0.91)	21.4% (0.83)
**Stand Up**	$F_{\mathrm{tot}}$	16.2% (0.96)	20.9% (0.94)
	$F_{\mathrm{med}}$	20.3% (0.94)	24.0% (0.89)
	$F_{\mathrm{lat}}$	16.2% (0.95)	23.0% (0.82)

## Data Availability

Data used in this study are publicly available and can be requested from https://cams-knee.orthoload.com.

## Abbreviations

The following abbreviations are used in this manuscript:

biLSTM-MLP	bidirectional Long Short-Term-Memory Network with a Multi-Layer Perceptron
TCN	Temporal Convolutional Network
KCF	Knee Contact Force
RMSE	Root Mean Squared Error
BW	Body Weight
EMG	Electromyography
KOA	Knee Osteoarthritis
TKA	Total Knee Arthroplasty
MSK	Musculoskeletal
ML	Machine Learning
LSTM	Long Short-Term-Memory
GRF	Ground Reaction Force
MLP	Multi-Layer Perceptron
ReLU	Rectified Linear Unit
$F_{\mathrm{med}}$	Medial Knee Contact Force
$F_{\mathrm{lat}}$	Lateral Knee Contact Force
$F_{\mathrm{tot}}$	Total Knee Contact Force
LOSO	Leave-One-Subject-Out
PCC	Pearson Correlation Coefficient
nRMSE	Normalized Root Mean Squared Error
LOTO	Leave-One-Trial-Out
LOFO	Leave-One-Feature-Out
CCI	Co-Contraction Index

## Appendix A

### Appendix A.1. LSTM Model: Effect of Input Normalization

Removing z-score normalization led to a consistent decline in model performance across all tasks and force components (Table A1). For example, for the $F_{\mathrm{tot}}$ during walking, the nRMSE rose from 11.9% to 31.2%, and PCC dropped from 0.97 to 0.38. Similar patterns were observed for $F_{\mathrm{med}}$ and $F_{\mathrm{lat}}$ predictions. These findings are aligned with established evidence that feature scaling significantly impacts machine learning model performance across various tasks and algorithms [32]. An example for the difference in performance between the normalized and non-normalized inputs can be seen in Figure A1.

**Table A1:** Comparison of model performance when using input features with and without z-score normalization. Values represent averages across subjects for each task and force component. Each cell contains the nRMSE (%), followed by the PCC in parentheses. Normalization significantly improved nRMSE and PCC across all conditions.

		Average Across Subjects (z-Score Normalized Inputs)	Average Across Subjects (Inputs Without z-Score Normalization)
**Walking**	$F_{\mathrm{tot}}$	11.9% (0.97)	31.2% (0.80)
	$F_{\mathrm{med}}$	11.9% (0.98)	34.7% (0.81)
	$F_{\mathrm{lat}}$	27.8% (0.66)	27.8% (0.27)
**Squat**	$F_{\mathrm{tot}}$	23.4% (0.84)	42.3% (0.09)
	$F_{\mathrm{med}}$	34.9% (0.61)	34.9% (0.24)
	$F_{\mathrm{lat}}$	17.8% (0.92)	47.7% (0.16)
**Downhill** **Walking**	$F_{\mathrm{tot}}$	13.1% (0.98)	34.8% (0.77)
	$F_{\mathrm{med}}$	13.3% (0.97)	35.3% (0.83)
	$F_{\mathrm{lat}}$	18.7% (0.90)	32.4% (0.44)
**Stairs** **Descent**	$F_{\mathrm{tot}}$	16.4% (0.96)	36.8% (0.75)
	$F_{\mathrm{med}}$	16.5% (0.95)	37.4% (0.71)
	$F_{\mathrm{lat}}$	20.5% (0.91)	35.2% (0.48)
**Sit Down**	$F_{\mathrm{tot}}$	17.6% (0.90)	35.3% (0.65)
	$F_{\mathrm{med}}$	22.1% (0.83)	31.8% (0.72)
	$F_{\mathrm{lat}}$	16.8% (0.91)	40.6% (0.61)
**Stand Up**	$F_{\mathrm{tot}}$	16.2% (0.96)	33.5% (0.44)
	$F_{\mathrm{med}}$	20.3% (0.94)	30.6% (0.51)
	$F_{\mathrm{lat}}$	16.2% (0.95)	39.7% (0.48)

### Appendix A.2. Impact of Kinematic Input Modalities

To assess the clinical applicability of our approach, we conducted additional LOFO analysis specifically examining different combinations of kinematic inputs. Table A2 presents model performance when excluding: (1) all kinematics (both marker-based and fluoroscopic), (2) only fluoroscopic kinematics (retaining marker-based), or (3) only skin marker kinematics (retaining fluoroscopic). The results show relatively modest differences between conditions, which is likely attributable to strong correlations between marker-based and fluoroscopic kinematic features, when one modality is removed, the other can partially compensate. Nevertheless, these findings suggest that acceptable prediction accuracy can be achieved using only marker-based motion capture systems, which are widely available in clinical gait laboratories, without requiring fluoroscopic imaging.

**Table A2:** LOFO validation results for different activities and force components. Reported values represent nRMSE (%) and PCC in parentheses, averaged across all cycles and subjects. “With all inputs” reflects the baseline model using all input features. Subsequent columns show performance when excluding either both kinematics data or only fluoroscopic or skin marker kinematics.

		With All Inputs	Without Kinematics	Without Fluoroscopic Kinematics	Without Skin Marker Kinematics
**Walking**	$F_{\mathrm{tot}}$	11.9% (0.97)	13.3% (0.96)	12.6% (0.96)	12.2% (0.96)
	$F_{\mathrm{med}}$	11.9% (0.98)	15.1% (0.96)	12.3% (0.97)	13.7% (0.97)
	$F_{\mathrm{lat}}$	27.8% (0.66)	20.7% (0.72)	22.3% (0.62)	21.0% (0.71)
**Squat**	$F_{\mathrm{tot}}$	23.4% (0.84)	31.0% (0.81)	21.5% (0.79)	23.3% (0.82)
	$F_{\mathrm{med}}$	34.9% (0.61)	37.2% (0.63)	31.3% (0.50)	32.5% (0.58)
	$F_{\mathrm{lat}}$	17.8% (0.92)	30.5% (0.89)	20.5% (0.88)	21.0% (0.89)
**Downhill** **Walking**	$F_{\mathrm{tot}}$	13.1% (0.98)	13.0% (0.97)	12.1% (0.98)	11.6% (0.98)
	$F_{\mathrm{med}}$	13.3% (0.97)	16.6% (0.96)	12.6% (0.97)	13.5% (0.97)
	$F_{\mathrm{lat}}$	18.7% (0.90)	19.1% (0.82)	18.0% (0.88)	16.7% (0.90)
**Stairs** **Descent**	$F_{\mathrm{tot}}$	16.4% (0.96)	17.6% (0.96)	14.3% (0.97)	14.7% (0.97)
	$F_{\mathrm{med}}$	16.5% (0.95)	18.7% (0.94)	13.9% (0.96)	16.4% (0.96)
	$F_{\mathrm{lat}}$	20.5% (0.91)	21.1% (0.84)	18.5% (0.90)	17.4% (0.91)
**Sit Down**	$F_{\mathrm{tot}}$	17.6% (0.90)	28.2% (0.87)	18.8% (0.91)	20.3% (0.89)
	$F_{\mathrm{med}}$	22.1% (0.83)	27.0% (0.84)	27.0% (0.73)	22.7% (0.83)
	$F_{\mathrm{lat}}$	16.8% (0.91)	30.5% (0.87)	20.2% (0.89)	23.7% (0.88)
**Stand Up**	$F_{\mathrm{tot}}$	16.2% (0.96)	20.3% (0.90)	18.2% (0.94)	13.4% (0.96)
	$F_{\mathrm{med}}$	20.3% (0.94)	22.3% (0.86)	19.1% (0.88)	18.1% (0.92)
	$F_{\mathrm{lat}}$	16.2% (0.95)	23.3% (0.87)	15.0% (0.94)	17.4% (0.95)
